# Airway Pressure Release Ventilation Mode Improves Circulatory and Respiratory Function in Patients After Cardiopulmonary Bypass, a Randomized Trial

**DOI:** 10.3389/fphys.2021.684927

**Published:** 2021-06-03

**Authors:** Huiqing Ge, Ling Lin, Ying Xu, Peifeng Xu, Kailiang Duan, Qing Pan, Kejing Ying

**Affiliations:** ^1^Department of Respiratory Care, Regional Medical Center for National Institute of Respiratory Diseases, Sir Run Run Shaw Hospital, School of Medicine, Zhejiang University, Hangzhou, China; ^2^Department of Critical Care Medicine, Sir Run Run Shaw Hospital, School of Medicine, Zhejiang University, Hangzhou, China; ^3^College of Information Engineering, Zhejiang University of Technology, Hangzhou, China; ^4^Department of Respiratory and Critical Care, Regional Medical Center for National Institute of Respiratory Diseases, Sir Run Run Shaw Hospital, School of Medicine, Zhejiang University, Hangzhou, China

**Keywords:** mechanical ventilation, airway pressure release ventilation, cardiopulmonary bypass, circulatory function, respiratory function

## Abstract

**Importance:**

Postoperative pulmonary complications and cardiovascular complications are major causes of morbidity, mortality, and resource utilization in cardiac surgery patients.

**Objectives:**

To investigate the effects of airway pressure release ventilation (APRV) on respiration and hemodynamics in post cardiac surgery patients.

**Main Outcomes and Measures:**

A single-center randomized control trial was performed. In total, 138 patients undergoing cardiopulmonary bypass were prospectively screened. Ultimately 39 patients met the inclusion criteria and were randomized into two groups: 19 patients were managed with pressure control ventilation (PCV) and 20 patients were managed with APRV. Respiratory mechanics after 4 h, hemodynamics within the first day, and Chest radiograph score (CRS) and blood gasses within the first three days were recorded and compared.

**Results:**

A higher cardiac index (3.1 ± 0.7 vs. 2.8 ± 0.8 L⋅min^–1^⋅m^2^; *p* < 0.05), and shock volume index (35.4 ± 9.2 vs. 33.1 ± 9.7 ml m^–2^; *p* < 0.05) were also observed in the APRV group after 4 h as well as within the first day (*p* < 0.05). Compared to the PCV group, the PaO2/FiO_2_ was significantly higher after 4 h in patients of APRV group (340 ± 97 vs. 301 ± 82, *p* < 0.05) and within the first three days (*p* < 0.05) in the APRV group. CRS revealed less overall lung injury in the APRV group (*p* < 0.001). The duration of mechanical ventilation and ICU length of stay were not significantly (*p* = 0.248 and 0.424, respectively).

**Conclusions and Relevance:**

Compared to PCV, APRV may be associated with increased cardiac output improved oxygenation, and decreased lung injury in postoperative cardiac surgery patients.

## Introduction

Postoperative pulmonary complications and cardiovascular complications are major causes of morbidity, mortality and resource utilization in cardiac surgery patients (Biccard et al., 2018). Patients undergoing cardiopulmonary bypass (CPB) frequently experience hypoxemia and pulmonary complications after surgery and may develop acute respiratory distress syndrome (Cox et al., 2000; Stephens et al., 2013). The passage of blood through the CPB circuit can activate inflammatory and coagulation pathways (Al Jaaly et al., 2015), which may lead to poor postoperative gas exchange and lung mechanics, an increase in the pulmonary shunt fraction and a reduction in functional residual capacity. Despite the advanced CPB techniques and the preventive measures used to avoid respiratory complications after cardiac surgery (Apostolakis et al., 2010; García-Delgado et al., 2014), postoperative acute respiratory distress syndrome manifests in 10-20% of patients (Gajic et al., 2011), and its overall mortality remains high (Habashi and Andrews, 2004; Kor et al., 2014). In addition, improper postoperative ventilator settings are associated with an increased risk for lung infection, a longer duration of intubation and a longer hospital stay (Wolthuis et al., 2008).

Airway pressure release ventilation (APRV) mode is a lung protective strategy that has been proposed to treat refractory hypoxemic respiratory failure while preventing ventilator-induced lung injury (Gary et al., 2017). APRV was originally described as a mode to treat acute lung injury in patients and attempt to maintain the level of airway pressure without reducing cardiac function, delivering mechanical breaths without excessive airway pressure and allowing unrestricted spontaneous ventilation. The potential benefits include decreased sedation, a shorter duration of mechanical ventilation, and an improvement in cardiac performance (Mireles-Cabodevila and Kacmarek, 2016). However, APRV introduces a higher mean airway pressure (Pmean) compared to conventional ventilation mode, which may increase intrathoracic pressure, and subsequently right atrial pressure. Hence, APRV might lead to a decrease in systemic venous return compared to conventional mechanical ventilation. Some studies have reported the effect of APRV on the respiratory and circulatory system in patients with ARDS, but there are few reports on the patients after cardiac surgery (Garner et al., 1988; Kaplan et al., 2001). We hypothesized that APRV could result in better outcomes in postoperative care of such population. A randomized controlled trial was designed to compare APRV with conventional PCV.

## Materials and Methods

### Study Design and Setting

This study protocol was approved by the Medical Ethics Committee of Sir Run Run Shaw Hospital and Zhejiang University School of Medicine (20181225-6). Written informed consent was obtained from all patients or their legal representatives prior to the study. The clinical trial number was ISRCTN92666776.

### Participants

**FIGURE 1:**
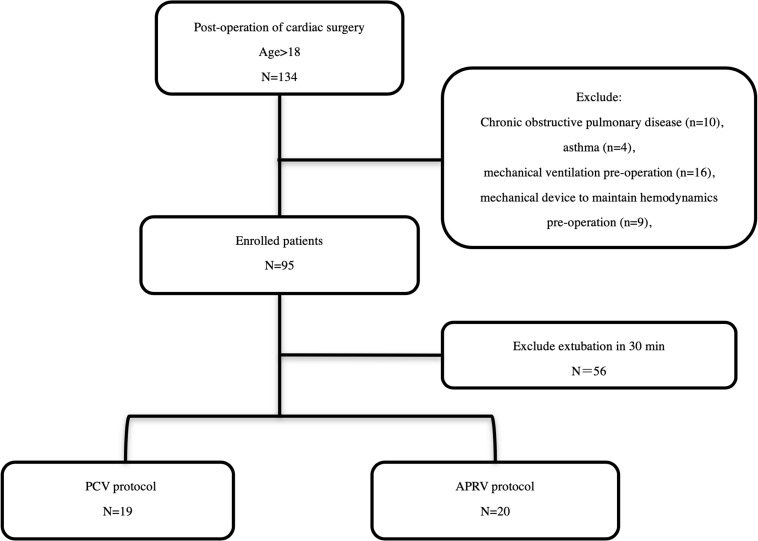
Study Enrollment and study protocol. In total, 138 patients were screened in the present study. Ninety-five patients were enrolled, and ultimately 39 patients were randomized.

Consecutive patients scheduled for cardiac surgery and afterward referred to the intensive care unit (ICU) from March 1st to May 31st, 2020 were included in the study. The exclusion criteria were age less than 18 years, history of chronic obstructive pulmonary disease, or asthma, mechanical ventilation prior to the operation, use of a mechanical device to maintain hemodynamics prior to the operation and extubation within 30 min after surgery (Figure 1). All patients were placed on mechanical ventilation. Patients who met all the inclusion criteria were randomly assigned to the PCV group or the APRV group Randomization was achieved with computer-generated random numbers, which were then stored in sealed envelopes.

### Anesthesia and Perioperative Management

Anesthetic technique was standardized. Endotracheal tubes and radial artery and pulmonary artery catheters were inserted after the induction of anesthesia. During anesthesia, patients were ventilated with volume-controlled ventilation (Fabius GS Premium, Germany) with the following settings: a tidal volume of 8 ml/kg, respiratory rate of 12 breaths per min, positive end-expiratory pressure (PEEP) of 5 cmH_2_O and fraction of inspired oxygen (FiO_2_) of 50%. Subsequently, the anesthesia was maintained with a continuous infusion of fentanyl and propofol, and rocuronium was used as a paralytic. The CPB was established via a standard median sternotomy, aortic root cannulation, and single or double atrial cannulation for venous return. CPB flow was kept between 2.2 and 2.5 l/min/m^2^ with a target mean perfusion pressure of 60 mmHg. If necessary, norepinephrine was administered to reach the targeted blood pressure. Weaning from CPB was initiated once the patient’s cardiac rhythm had stabilized and normothermia had been achieved. Standard clinical postoperative care with goal-directed therapy was initiated after separating the patient from the CPB machine and was continued during the ICU stay to maintain hemodynamic stability. The central venous pressure (CVP) was kept between 10 and 12 mmHg and pulmonary artery wedge pressure (PAWP) between 15 and 18 mmHg by the administration of intravenous fluids. Inotropic support with epinephrine was initiated in order to maintain a cardiac index (CI) greater than 2.2 l/min/m^2^. Milrinone was administered if epinephrine was increased to a maximum of 0.1 μg/kg/min and additional inotropy was needed. The target mean arterial blood pressure was set at >65 mmHg and norepinephrine was administered if necessary. Lactate was targeted at <2 mmol/L and mixed venous oxygen saturation (SvO2) at >70%. An intra-aortic balloon pump was inserted if further support was required. Hemoglobin was maintained at >70 g/L with transfusion of packed red blood cells. The decision to weaning patients from inotropic and vasopressor medications was made by the attending ICU physicians based on the assessment of hemodynamic data, urine output and physical status. These physicians were blinded in our study. Pain medication was used in all patients to maintain a pain scale score less than three points, as measured by a numerical rating scale or the Critical Care Pain Observational Tool (Rijkenberg et al., 2015). Patients were discharged from the ICU when the following criteria were met: stable hemodynamics without any vasoactive agents, peripheral capillary oxygen saturation (SpO2) >94% at an FiO_2_ <0.5 by facemask, chest tube drainage <200 mL within the past 8 h, and urine output >0.5 mL/kg/h.

### Ventilation Protocol

#### Baseline Settings

After admission to the ICU, patients were connected to Evita 4 ventilators (Dräger Medical, Lübeck, Germany). All patients were ventilated with volume-controlled ventilation in assist control mode for the first 30 min. The tidal volume was set to 6–8 ml/kg per kilogram of predicted body weight according to the plateau pressure (Pplat) (to maintain Pplat <30 cm H_2_O); the Pplat measured during conventional ventilation by using the ventilator’s inspiratory pause control. The levels of PEEP and FiO_2_ were based on the patient’s underlying clinical condition to obtain an SpO2 of 90%–95% or an arterial partial pressure of O2 (PaO_2_) of 60–80 mmHg. The respiratory rate was set to maintain a partial pressure of arterial carbon dioxide (PaCO_2_) between 35 and 45 mmHg. A stepwise maximum alveolar recruitment maneuver (RM) was performed in all patients.

#### RM Process

This process was accomplished as follows: the mechanical ventilator was set to PCV mode with FiO_2_ of 100%, a respiratory rate of 12/min and an I: E ratio of 1:1. An incremental PEEP trial was performed starting at a pressure of 10 cmH_2_O with steps of 5 cmH_2_O and a duration of 1 min per step until the peak pressure reached 45 cmH_2_O. The driving pressure was set to 15 cmH_2_O. The duration of the highest step was 2 min instead of 1. The RM was stopped if one or more of the following signs of clinical deterioration were observed: heart rate ≥140 or <60 bpm; MBP <65 mmHg or systolic blood pressure <90 mmHg; SpO2 <88% or 5% decreased from baseline; or acute atrial fibrillation, atrial flutter or ventricular tachycardia (Dean, 2015).

#### Ventilation in the PCV Group

After the RM, the driving pressure was adjusted to match the value at baseline. Appropriate adjustments to the respiratory rate were made to maintain PaCO_2_ between 35 and 45 mmHg. The PEEP and FiO_2_ were adjusted in response to changes in the SpO2 along the “Lower PEEP/Higher FiO_2_” scale (The Acute Respiratory Distress Syndrome Network, 2000; Kasenda et al., 2016). The Pplat was kept below 30 cmH_2_O.

#### Ventilation in the APRV Group

In the APRV group, the ventilator mode was switched to APRV with the following initial settings: The PHigh was initially set to the Pplat at baseline. The PLow was set to 0 cmH_2_O. The duration of the PHigh (THigh) was set to 3.6–6.0 s (minimal 90% of the total respiratory cycle time). The duration of the release phase (TLow) was set to terminate at 75% of the peak expiratory flow rate. This parameter was calculated and monitored for adjustment based on the angle of deceleration noted on the expiratory flow waveform. The PHigh, THigh, TLow and FiO_2_ were titrated based on interpretation of the expiratory flow waveform and arterial blood gasses (Garner et al., 1988; Habashi, 2005).

### Cardiovascular Measurements

Heart rate, mean arterial systemic blood pressure (MAP), CVP, mean pulmonary artery pressure (PAP), and PAWP, end diastolic velocity (EDV), pulmonary vascular resistance index (PVRI), cardiac output (CO), CI, and stroke volume (SV) were measured (intelivue MP60, Philips, Netherlands) and recorded (Swan-Ganz, Arrow Company, United States). The Swan-Ganz catheter was removed after the first ICU day after hemodynamics stability was confirmed.

### Respiratory Mechanics and ABG Measurements

The baseline respiratory mechanics were recorded at once the patients admitted to ICU and connected to the ventilator (T0), 1 h after mechanical ventilation (T1), and 4 h after mechanical ventilation (T2). Dynamic lung mechanics were calculated by the ventilators. PEEPi measurement was performed with end-expiratory occlusion. The Pplat was measured with a breath holding technique at the end of inspiration when the patients were sedated maintained Richmond at a Agitation Sedation Scale (Rass) score of −2. The mean airway pressure was calculated by the ventilator as the geometric mean of the pressure-time curve. Arterial blood gasses were examined every 4 h on the first day and once every morning on days 2–3 (GEM Premier 3000, IL, United States). The central laboratory of Sir Run Run Shaw Hospital performed clinical pathology tests and blood cultures.

### Chest Radiograph Score

Chest radiograph score (CRS) (Antonio et al., 2005) were obtained pre-operatively and 1 h, 1 day, and 3 days postoperatively. Anterior chest radiographs were assessed (Model X-ray tube assembly 0.7/1.3U163C-36, SHIMADZU Corporation, Japan) using a standardized technique (70 kV, 3.8 mAs, 100 cm film-focus distance for anteroposterior; broad tube focus for both). Each lung zone was evaluated by scoring the radiographs with a four-point scale based on visual assessment (Antonio et al., 2005; Habashi, 2005) as follows: 0 = normal (no alveolar consolidation), 1 = alveolar consolidation confined to 1 quadrant, 2 = alveolar consolidation confined to 2 quadrants, and 3 = alveolar consolidation confined to 3 quadrants, 4 = alveolar consolidation in 4 quadrants. Radiologists independently evaluate chest x-ray. In addition to the information evident on the radiographs, the radiologists were blinded to the group assignment, clinical progress and final outcomes of the patients.

### Data Collection and Statistical Analysis

The primary endpoints of the study were the effects of APRV on cardiorespiratory function, including lung mechanics, blood gasses and CRS within 3 days, and hemodynamic parameters within 1 day. The secondary endpoint was the duration of mechanical ventilation and ICU stay.

Data were tested for normal distribution with the Shapiro–Wilks *W*-test. The results were expressed as the means ± standard deviations. For blood gasses and hemodynamics, data were analyzed by two-way analysis of variance (ANOVA), with the ventilation mode and time after randomization as independent variables. Differences between the means were analyzed separately with a post hoc Newman–Keuls multiple comparison test. Baseline demographics and respiratory mechanics of the two groups were compared using two-tailed unpaired Student’s t-tests or chi-squared tests, where applicable. Differences were considered statistically significant for results with *p* < 0.05.

## Results

### Patient Characteristics

In total, 134 patients were screened in the present study. Ninety-five patients were enrolled, and ultimately 39 patients were randomized (Figure 1). The types of surgery were: mitral valve replacements (26/39), tricuspid valve repairs (5/39), aortic valve replacements (2/39), coronary artery bypass graft (5/39), and cardiac tumor resection (1/39). The mean age was 56.0 ± 14.7 years, male/female was 18/21. The baseline characteristics of the patients are summarized in Table 1. No statistically significant differences were found between the two study groups at baseline.

**TABLE 1:** Patient demographics and baseline parameters.

Parameter	APRV (*N* = 20)	PCV (*N* = 19)	*p*-value
Age, year	55 ± 15	58 ± 15	0.45
Sex, M/F	11/9	7/12	0.26
APACHE II	14 ± 5	17 ± 8	0.24
EuroSCORE II	7.5 ± 4.4	7.4 ± 8.2	0.98
Disease, *n* (%)			
Valve repair or replacement	17 (85)	16 (84)	0.99
CABG	2 (10)	3 (15)	0.95
Resection of a cardiac tumor	1 (5)	0 (0)	
Smoking, *n* (%)			
Yes	12 (60)	11 (57.9)	0.89
No	8 (40)	8 (42.1)	
Operation time, min	261.4 ± 86.6	238.4 ± 94.4	0.43
CPB time, min	139.2 ± 67.3	119.4 ± 71.3	0.85
Clamping time, min	101 ± 48.6	81.1 ± 45.0	0.89
Cst (ml/cmH_2_O)	43.4 ± 13.5	42.7 ± 7.1	0.84
R (cmH_2_O/(L⋅s))	12.7 ± 3.6	8.9 ± 1.4	0.32

*APACHE, Acute Physiology and Chronic Health Evaluation II; CABG, coronary artery bypass grafting; CPB, cardiopulmonary bypass; Cst, static compliance; R, resistance. Values are expressed as means ± SD.*

### Hemodynamics and Laboratory Data

The APRV and PCV groups demonstrated hemodynamic stability during mechanical ventilation. MAP, MPAP, and CVP changes did not differ between groups at any measurement time points (Table 2). The increase in the CI and SI were significantly higher with APRV than with PCV after 4 h (*p* = 0.017 and *p* = 0.024, respectively). PVRI and right ventricular work (RVSW) were lower in the APRV group (*p* = 0.028 and *p* = 0.002, respectively).

**TABLE 2:** Hemodynamics parameters.

Variables	Group	Baseline	4 h	8 h	1 d	*p*-value
HR (beats/min)	PCV	85.8 ± 9.9	86.1 ± 11.6	87.2 ± 9.6	85.7 ± 10.0	0.308
	APRV	90.0 ± 13.7	90.6 ± 13.7	88.4 ± 11.9	85.0 ± 14.2	
MAP (mmHg)	PCV	85 ± 11	79 ± 11	74 ± 6	73 ± 6	0.743
	APRV	81 ± 11	77 ± 9	78 ± 7	79 ± 9	
MPAP (mmHg)	PCV	24.9 ± 9.1	22.4 ± 5.5	23.1 ± 4.4	24.1 ± 4.8	0.527
	APRV	26.2 ± 5.5	23.6 ± 4.6	23.3 ± 4.7	24.2 ± 5.7	
CVP (mmHg)	PCV	11.1 ± 2.6	9.9 ± 3.4	11.5 ± 2.8	11.3 ± 2.8	0.59
	APRV	10.6 ± 2.0	10.4 ± 2.6	10.1 ± 2.1	12.3 ± 2.4	
CI (L ⋅ min^–1^ ⋅ m^2^)	PCV	2.8 ± 0.8	2.8 ± 0.8	2.5 ± 0.7^#^	2.7 ± 0.4	0.017
	APRV	2.8 ± 0.7	3.1 ± 0.7*^#^	2.9 ± 0.5*	3.1 ± 0.5*^#^	
SI (ml ⋅ m^–2^)	PCV	32.6 ± 10.0	33.1 ± 9.7	29.0 ± 9.2	31.7 ± 7.7	0.024
	APRV	33.3 ± 7.1	35.4 ± 9.2*^#^	35.3 ± 7.4*^#^	37.2 ± 6.4*^#^	
PVRI (dyne ⋅ s ⋅ cm^–5^ ⋅ Min ⋅ m^2^)	PCV	338.4 ± 169.5	339.2 ± 179.4	296.5 ± 85.3^#^	286.9 ± 125.1^#^	0.028
	APRV	315.1 ± 147.3*	248.3 ± 91.3*^#^	222.2 ± 100.3*^#^	215.1 ± 105.0*^#^	
SVRI (dyne ⋅ s⋅ cm^–5^ ⋅ Min ⋅ m^2^)	PCV	2334 ± 843	2079 ± 826	2165 ± 499	1901 ± 347	0.096
	APRV	2258 ± 478	1814 ± 527	1903 ± 398	1851 ± 500	
PAWP (mmHg)	PCV	14.2 ± 5.2	12.6 ± 2.9	14.3 ± 3.7	14.8 ± 3.0	0.007
	APRV	15.6 ± 4.9	15.1 ± 4.6	15.8 ± 3.8	16.2 ± 3.6	

HR, heart rate; MAP, mean arterial pressure; MPAP, mean pulmonary arterial pressure; CVP, central venous pressure; CI, cardiac index; SI, shock volume index; PVRI, pulmonary vascular resistance index; SVRI, systemic vascular resistance index; PAWP, pulmonary artery wedge pressure. Values are expressed as means ± SD. *p < 0.05 APRV vs. PCV at the same time; ^#^p < 0.05 vs. baseline in the same group.

### Pulmonary Mechanics and Gas Exchange

Most of the patients (85.7%) were extubated before 8 h. The compliance and resistance were calculated by the ventilator with using least squares Regression. The ID of the endotracheal tube was 8.0 mm for males and 7.5 mm for females. There was no significant difference between the PCV and APRV groups in lung compliance and airway resistance or other baseline pulmonary characteristics. Patients in the PCV group had a higher peak airway pressure (PCV vs. APRV, 15.7 ± 2.1 vs. 18 ± 2.2, *p* = 0.000) and Pplat (PCV vs. APRV, 18.5 ± 2.9 vs. 16.3 ± 3.8, *p* = 0.000) after randomization. The Pmean was significantly higher in the APRV group than in the PCV group after transition from assist-control ventilation (*p* = 0.000) (Table 3). Cst after 4 h was higher in the APRV group (*p* = 0.001, Table 3).

**TABLE 3:** Ventilator settings after randomization after 4 h and respiratory mechanics after 1 and 4 h.

Variables	Group	Baseline	4 h	8 h	*p*-value
Cstat (ml/cmH_2_O)	PCV	42 ± 7	49 ± 11	41 ± 8	0.008
	APRV	43 ± 9	49 ± 9^#^	52 ± 11*^#^	
Rinsp (cmH_2_O)/(L⋅s)	PCV	9 ± 1	10 ± 2	7 ± 2	0.302
	APRV	9 ± 2	13 ± 9	8 ± 2	
Pplat (cmH_2_O)	PCV	19 ± 3	18 ± 3	18 ± 3	0.004
	APRV	16 ± 3*	16 ± 3*	15 ± 3*	
P_mean_ (cmH_2_O)	PCV	9 ± 2	9 ± 3	9 ± 2	0.002
	APRV	11 ± 3*	13 ± 2*	11 ± 2*	
Ppeak (cmH_2_O)	PCV	18 ± 2	18 ± 3	19 ± 3	0.000
	APRV	15 ± 2*	16 ± 3*	15 ± 3*	
PEEPtotal (cmH_2_O)	PCV	6 ± 2	6 ± 2	6 ± 1	0.075
	APRV	5 ± 1	5 ± 1	5 ± 1	
DP (cmH_2_O)	PCV	13 ± 2	14 ± 2	13 ± 2	0.000
	APRV	12 ± 2	10 ± 3*	10 ± 5*	
VT (L)	PCV	0.48 ± 0.06	0.50 ± 0.08	0.49 ± 0.08	0.457
	APRV	0.48 ± 0.07	0.47 ± 0.08	0.49 ± 0.11	

Ppeak, peak pressure; Pplat, plateau pressure; Pmean, mean airway pressure; PEEP, positive end-expiratory pressure; VT, tidal volume; RR, respiratory rate in beat per minute; Cst, static compliance; R, resistance. Values are expressed as means ± SD. *p < 0.05 APRV vs. PCV at the same time; ^#^p < 0.05 vs. baseline in the same group.

A higher PaO_2_/FiO_2_ (*p* = 0.002) and central venous oxygen saturation (ScvO_2_, *p* = 0.013) were found in the APRV group compared to the PCV group after 4 h. Arterial pH, PaCO_2_, and SaO_2_ were comparable in the two groups (Table 4). The lactate level was higher in the PCV group (*p* = 0.000). CRS was improved in the APRV group compared to the PCV group (*p* = 0.001, Table 5).

**TABLE 4:** Arterial Blood gasses analysis.

Variables	Group	Baseline	4 h	8 h	1 days	2 days	3 days	*p*
PH	PCV	7.36 ± 0.10	7.31 ± 0.07	7.31 ± 0.06	7.32 ± 0.05	7.36 ± 0.06	7.40 ± 0.03	0.785
	APRV	7.35 ± 0.08	7.34 ± 0.06	7.31 ± 0.07	7.34 ± 0.08	7.34 ± 0.04	7.38 ± 0.05	
PaCO_2_ (mmHg)	PCV	35 ± 12	35 ± 7	35 ± 7	37 ± 8	45 ± 9^#^	43 ± 6^#^	0.030
	APRV	39 ± 8	36 ± 5	38 ± 4	38 ± 6	47 ± 10^#^	44 ± 5^#^	
HCO_3_ (mmol/L)	PCV	20 ± 4	18 ± 4	17 ± 4	19 ± 5	24 ± 3	24 ± 2	0.074
	APRV	21 ± 2	19 ± 3	19 ± 3	20 ± 3	24 ± 3	25 ± 4	
P/F ratio	PCV	284 ± 86	301 ± 82	312 ± 76	292 ± 84	231 ± 62	268 ± 55	0.002
	APRV	292 ± 86	340 ± 97*^#^	352 ± 81*^#^	334 ± 74*^#^	284 ± 93*	306 ± 71*	
P (A-a) DO_2_ (mmHg)	PCV	131 ± 48	103 ± 55	107 ± 46	110 ± 50	132 ± 47	107 ± 38	0.162
	APRV	121 ± 40	104 ± 47	90 ± 38	101 ± 42	115 ± 63	97 ± 51	
Lactate (mmol/L)	PCV	5.22 ± 3.54	6.76 ± 3.43	7.45 ± 3.67	6.36 ± 4.24	3.06 ± 1.72	2.05 ± 1.30	0.000
	APRV	3.18 ± 2.77	4.23 ± 2.67	5.32 ± 3.74	4.59 ± 3.46	2.58 ± 0.80	1.63 ± 0.67	
ScVO_2_ (%)	PCV	58 ± 14	56 ± 13	50 ± 16	49 ± 8	61 ± 6	66 ± 5^#^	0.013
	APRV	60 ± 13	64 ± 12*^#^	61 ± 12*	58 ± 9*	66 ± 6*^#^	70 ± 4*^#^	

*Values are expressed as means ± SD. ^∗^p < 0.05 APRV vs. PCV at the same time; ^#^p < 0.05 vs. baseline in the same group. P/F ratio = PaO_2_/FiO_2_ ratio.*

**TABLE 5:** Chest radiograph score (CRS).

Variables	Group	Baseline (pre-operation)	1 day	2 days	3 days	*p*
CRS (%)	PCV	0.6 ± 0.9	1.2 ± 0.7	1.6 ± 0.8	1.1 ± 0.8	0.001
	APRV	0.4 ± 0.7	0.8 ± 0.8	0.6 ± 0.8	0.4 ± 0.8	

*CRS, Chest radiographic scores. Values are expressed as means ± SD.*

### Mechanical Ventilation Time and ICU Stay

The duration of mechanical ventilation was lower, but not significantly lower, in the APRV groups (14.3 ± 16.0 vs. 18.4 ± 20.7 h, APRV vs. PCV, respectively; *p* = 0.248). The ICU length of stay was not significantly different (59.5 ± 39.3 vs, 79.6 ± 52.6 h, APRV vs. PCV, respectively; *p* = 0.424) (Table 6).

**TABLE 6:** Mechanical ventilation time.

Parameter	APRV	PCV	*t*	*p*-value
MV Time (h)	14.3 ± 16.0	18.4 ± 20.7	0.699	0.248
ICU length of stay(h)	59.5 ± 39.3	79.6 ± 52.6	1.357	0.424

*Values are means ± SD.*

## Discussion

Airway pressure release ventilation made effects of lung mechanics, hemodynamics and blood gasses in post cardiac surgery patients. Compared with PCV, we found that APRV may be associated with higher Cst, PaO_2_/FiO_2_ and ScvO_2_, increased CI and improved CRS. This study is the first randomized trial demonstrating the potential benefit of APRV in patients after cardiac surgery.

Right ventricular (RV) failure post cardiac surgery increases morbidity and mortality (Bhama et al., 2018). Lung collapse induced by general anesthesia and CPB may increase RV outflow impedance by activation of the hypoxic pulmonary vasoconstriction reflex and by the geometrical changes of pulmonary capillaries with atelectasis, regardless of mild local hypoventilation or complete atelectasis (Brismar et al., 1985; Moudgil et al., 2005). In our study, cardiac surgery resulted in increased PVR and decreased PA compliance, both known to negatively affect RV performance. Since high PAP and PVR significantly contribute to RV dysfunction, it seems reasonable to apply a ventilation strategy using APRV mode to reverse pulmonary atelectasis and minimize both PAP and PVR to protect both the lungs and the right ventricle.

Classic physiology states that PVR has a U-shaped relationship with lung volume, its lowest value corresponding to functional residual capacity. According to Shekerdemian (Shekerdemian and Bohn, 1999), PVR, the major determinant of RV afterload, is related to lung volume in a bimodal fashion. The total resistance of the pulmonary circulation depends on the balance in the vascular tone of its two components: the alveolar vessels and the extra-alveolar or parenchymal vessels. Furthermore, atelectasis gives rise to intrapulmonary shunting (perfused but non-ventilated lung regions) and subsequent hypoxia. Overdistension, on the other hand, causes dead space ventilation (ventilated but non-perfused lung regions), leading to hypercapnia. Both hypercapnia and hypoxia increase PVR and RV afterload (Lynch et al., 1979; Mekontso Dessap et al., 2009). In our study, in the APRV group, PVR decreased significantly compared to the PCV group (*p* < 0.05). APRV may ameliorate lung collapse, increases lung volume and decreases PVR.

Numerous animal and clinical studies have shown that venous return is often sustained during ventilation with PEEP (Jansen and Versprille, 1986; Johnson and Normann, 1989). Potentially, PEEP-induced diaphragmatic descent increases abdominal pressure. van den Berg and Pinsky (2002) found that, in hemodynamically stable fluid-resuscitated postoperative surgical patients, inspiratory-hold maneuvers with a Pplat increase up to 20 cm H_2_O have minimal effects on CO, primarily because during the inspiratory phase, the abdominal compartment is pressurized causing compression of the liver and the lungs. Thus, with normal blood volume, increased intrathoracic pressure in a certain range does not lead to a significant decrease in blood pressure. In our research, standard clinical postoperative care with goal-directed therapy was used to maintain hemodynamic stability. Therefore, despite the higher mean airway pressure in the APRV group, the MAP and MPAP were similar to those in the PCV group. The CI was higher in the APRV group (Table 2). During the treatment, the Phigh setting was titrated with MAP and oxygen. After cardiac surgery, PVR increased in patients who developed lung collapse and pulmonary oedema. Cardiopulmonary interaction ensures that with the right intrathoracic pressure and appropriate tidal volume, the lowest PVR can be reached. At the same time, increased intrathoracic pressure reduces left ventricular afterload. These combined factors are beneficial in increasing the CI.

Spontaneous breathing was associated with an increase in CI. This finding is in agreement with the concept that a decrease in intrathoracic pressure during spontaneous inspiration with APRV may improve venous return and CI (Lee et al., 2017). Our protocol targeted CVP and PAWP as well. In our patients, SVO2 were improved as a result of improved CI. Increased oxygen delivery with unchanged oxygen consumption resulted in an improved relationship between tissue oxygen supply and demand, which may also have contributed to the higher PaO_2_ during APRV with spontaneous breathing.

### The Effect of APRV on Respiratory Mechanics

Airway pressure release ventilation maintains a sustained Paw over a large proportion of the respiratory cycle, and therefore this ventilation strategy has a high pressure-time profile (Lynch et al., 1979). The pathologic tetrad of permeability, edema, surfactant deactivation and alveolar instability are the early drivers of postoperative lung pathology in the setting of cardiac surgery. We hypothesized the early introduction of APRV would specifically target these key elements of post cardiac surgery lung pathophysiology and prevent lung function injury. The recruitment of alveolar units is known to be a function of both ventilator pressure and time (Jansen and Versprille, 1986). APRV had a significantly higher Pmean than PCV due to the extended time at the Phigh, and had a lower Ppeak and Pplat than PCV (Table 3). Compared to the application of PCV with higher PEEP, which requires a tidal volume above PEEP in order to ventilate, APRV described as CPAP with a brief release, releases pressure from the Phigh in order to generate a ventilatory volume. APRV can harness the potential energy contained within the elastic properties of the respiratory system that cause the lung to recoil naturally to generate the tidal volume (Neumann et al., 2005; Güldner et al., 2014). The data suggest that rather than over-distending alveoli, the extended THigh/PHigh redistributes gas from the alveolar ducts to the alveoli, where it belongs (Schiller et al., 2001; Santos et al., 2005), and converts heterogeneous alveolar ventilation to homogeneous alveolar ventilation (Seah et al., 2011). Thus, the extended inspiratory duration has a powerful positive impact on reducing strain at the alveolar level.

Transpulmonary pressure could be used to evaluate the driving pressure and confirm the appropriate parameters settings. Due to the financial issues and the invasiveness of the operation, transpulmonary pressure was not routinely monitored, which was a limitation of the present study. The driving pressures of the two groups were the same, but the area under the pressure-time curve, which is defined as pressure/time profile (P/Tp), was different (Roy et al., 2013). APRV maintains a sustained airway pressure over a large proportion of the respiratory cycle, and therefore, this ventilation strategy has a higher P/Tp, which improved alveolar recruitment, reduced oedema and subsequently improved oxygenation (improved the P/F ratio). Early intervention using a ventilator mode with a high P/TP was recommended to prevent ARDS (MacDonnell et al., 2012; Roy et al., 2013).

Chest radiograph score assesses the extent of pulmonary exudative lesions by bilateral lung infiltration images. Our study found CRS was lower in the APRV group compared to PCV group, suggesting that the range of exudative lesions is smaller.

Limitations of our study include the fact that the number of patients enrolled was not large enough for stratification analysis. The baseline difference in the lactate level could have been caused by the small sample size. Our current data did not suggest the application of APRV would have significantly reduced the MV and ICU length of stay. Limited sample size in the present study could be one of the reasons for this finding. Urine output was not recorded after cardiac surgery. Although urine output is a non-invasive, primary variable for understanding the CO, it is often insufficient for hemodynamic evaluation, rapid assessment, and the identification of occult or compensated shock (Suess and Pinsky, 2015). The duration of ventilation was shorter in the APRV group but not significantly shorter. Since no previous studies provided information regarding the differences between APRV and PCV, we could not accurately calculate the effective sample size. The post hoc power for lung mechanics, P/F ratio and CRS indicated varied from 27.4% (P/F ratio at 4 h) to 78.0% (CRS at day 3). With the current findings we hope to provide information for *a priori* sample size calculation for future multi-centered randomized control trials.

In conclusion, compared to PCV, APRV may be associated with increased CO, improved oxygenation, and decreased lung injury after post operation of cardiac surgery.

## Data Availability Statement

The original contributions presented in the study are included in the article/supplementary material, further inquiries can be directed to the corresponding author/s.

## Ethics Statement

The studies involving human participants were reviewed and approved by the Ethics Committee of Sir Run Run Shaw hospital. The patients/participants provided their written informed consent to participate in this study.

## Author Contributions

HG and KY designed the study. HG and LL drafted the manuscript with input from all authors. YX, PX, and KD were responsible for collecting clinical data. QP analyzed the data with input from all authors. All authors contributed to the interpretation of the data and approved the final of the manuscript.

## Conflict of Interest

The authors declare that the research was conducted in the absence of any commercial or financial relationships that could be construed as a potential conflict of interest.

## Abbreviations

CPB: cardiopulmonary bypass
APRV: Airway pressure release ventilation
Pmean: airway mean pressure
PCV: pressure control ventilation
ICU: Intensive care unit
FiO_2_: fraction of inspired oxygen
CVP: Central venous pressure
PAWP: pulmonary artery wedge pressure
SvO_2_: mixed venous oxygen saturation
SpO_2_: peripheral capillary oxygen saturation
P_*plat*_: plateau pressure
PaO_2_: arterial partial pressure of O_2_
PaCO_2_: partial pressure of arterial carbon dioxide
RM: alveolar recruitment maneuver
CRS: Chest radiographic scores
PVR: pulmonary vascular resistance
DO_2_: oxygen delivery
VO_2_: oxygen consumption.
